# Human exome sequence data in support of somatic mosaicism in carotid atherosclerosis

**DOI:** 10.1016/j.dib.2021.107656

**Published:** 2021-12-01

**Authors:** Alexei A. Sleptcov, Alexei A. Zarubin, Polina M. Bogaychuk, Mikhail S. Kuznetsov, Boris N. Kozlov, Maria S. Nazarenko

**Affiliations:** a Research Institute of Medical Genetics, Tomsk National Research Medical Center, 10 Ushayka Embankment, Tomsk 634050, Russia; b Cardiology Research Institute, Tomsk National Research Medical Center, 111-A Kievskaja Str, Tomsk 634012, Russia; c National Research Tomsk State University, 36 Lenin Ave, Tomsk 634050, Russia

**Keywords:** Atherosclerosis, Carotid plaque, Exome sequencing, HiSeq

## Abstract

Understanding the mechanisms underlying the connection between somatic mosaicism and cardiovascular disease is likely essential for the future of personalized medicine. This article is aimed at providing data on somatic mosaicism in human carotid atherosclerosis. An advanced carotid atherosclerotic plaque and white blood cells were collected simultaneously from each patient (eight Slavic males, aged 67 ± 3.8 years [mean ± SD]) to assess the spectrum of germline and somatic genetic variants. Exome sequencing of DNA from the samples was performed with the SureSelect Clinical Research Exome Enrichment Kit (Agilent Technologies) and HiSeq 1500 (Illumina). The dataset contains germline and somatic single-nucleotide variants and small indels identified in the advanced carotid atherosclerotic plaque and white blood cells of each patient. This dataset does not include copy number variants owing to a lack of suitable tools for reliable calculation of copy numbers from exome sequencing data on cancer-unrelated samples. The dataset should help to understand somatic mosaicism in cardiovascular diseases and to identify copy number variants by means of more appropriate newer tools in the future.

## Specifications Table

**Table:** 

Subject	Genetics: Human
Specific subject area	Genetic Susceptibility to Atherosclerosis
Type of data	Table
How the data were acquired	Exon regions were sequenced via HiSeq 1500 (Illumina) with an average 150 × depth of coverage. The SureSelect Clinical Research Exome V2 Exome Enrichment Kit (Design ID S06588914) was used following the manufacturer's instructions (Agilent Technologies). The data on germline and somatic single-nucleotide polymorphisms (SNPs) and small indels were obtained by means of the Genome Analysis Toolkit (GATK) with Best Practices Workflows.
Data format	Raw Analyzed
Description of data collection	Total-genomic-DNA samples were extracted from white blood cells and advanced-carotid-atherosclerotic-plaque specimens with the DNeasy Blood and Tissue Kit (Qiagen). Library preparation was performed using the SureSelect XT2 (SSXT2) Reagent Kit and the SureSelect Clinical Research Exome V2 Exome Enrichment Kit (Design ID S06588914, Agilent Technologies). Sequencing was carried out on the Illumina HiSeq 1500 platform.
Data source location	Research Institute of Medical Genetics, Tomsk National Research Medical Center, Tomsk, Russia.
Data accessibility	Repository name: NCBI BioProject Data identification number: PRJNA758796 Direct URL to data: https://www.ncbi.nlm.nih.gov/bioproject/758796 Analyzed data Repository name: Mendeley Data Sleptcov, Alexei; Zarubin, Alexei (2021), “Germline SNPs and indels in patients with atherosclerosis”, Mendeley Data, V1, doi:10.17632/hj68dfm5sm.1https://data.mendeley.com/datasets/hj68dfm5sm/draft?a=accc8cd7-b87c-4dfb-a159-3ac356ef87f3 Sleptcov, Alexei; Zarubin, Alexei (2021), “Somatic SNPs and indels in patients with atherosclerosis”, Mendeley Data, V1, doi:10.17632/2byy5b3g6y.1 https://data.mendeley.com/datasets/2byy5b3g6y/draft?a=869efe83-ac48-4737-a447-d7bfe70dcf69 Clinical information on the patients Repository name: Mendeley Data Sleptcov, Alexei (2021), “Clinical information of patients with atherosclerotic plaque: exome sequenced”, Mendeley Data, V1, doi:10.17632/bwpb4jjf3j.1 https://data.mendeley.com/datasets/bwpb4jjf3j/draft?a=2aae82aa-1904-4e45-a77b-dd9fec672434

## Value of the Data

• The data on both germline and somatic mutations associated with carotid atherosclerosis can serve as the basis for studies on its pathogenesis and searches for biomarkers of disease severity and for new therapeutic targets.
• The dataset should be useful for identifying copy number variants with more appropriate newer tools in the future.
• The data can be helpful for further research into somatic mosaicism in atherosclerosis and clonal expansion in noncancerous tissues.
• The data can be integrated with other multi-omics studies or existing databases to better understand molecular mechanisms of complex traits and diseases.

## 1 Data Description

Clinical information about the patients with advanced carotid atherosclerosis is presented in Mendeley Data and contains the following characteristics: ID, age, sex, height, weight, body–mass index (BMI), waist circumference (WC), ischemic heart disease (IHD) symptoms, comorbidities, atherosclerotic-plaque description, and echocardiogram data. All patients had coronary artery disease in their medical history, abdominal obesity, arterial hypertension, hypercholesterolemia, and class II of heart failure (New York Heart Association).

All exomes were sequenced using the Illumina HiSeq platform in 2  ×  150 bp paired-end format. The dataset provides raw data, alignment data, and called and filtered data on both white blood cells and an advanced carotid atherosclerotic plaque collected from each patient (eight patients).

FASTQ raw data files and BAM alignment data files (hg19) were deposited in the NCBI database under BioProject database number PRJNA758796 (https://www.ncbi.nlm.nih.gov/bioproject/758796). The data cover eight patients, each of them has two BioSamples: white blood cells and the carotid atherosclerotic plaque (Table 1). Each BioSample accession is associated with SRA accessions linked with FASTQ raw data files and BAM alignment data files.

**Table 1:** Data structure in BioProject PRJNA758796 (patients with advanced carotid atherosclerosis).

Patient ID	Tissue type	BioSample Accession No.	Size of .fastq, GB*	Size of .bam, GB
#46	Leukocytes	SAMN21035664	2.74	4.68
	Plaque	SAMN21035748	3.64	5.94

#57	Leukocytes	SAMN21035741	4.24	6.59
	Plaque	SAMN21035749	3.63	5.92

#60	Leukocytes	SAMN21035742	4.14	6.48
	Plaque	SAMN21035750	4.09	6.72

#67	Leukocytes	SAMN21035743	4.06	6.40
	Plaque	SAMN21035751	3.76	6.14

#95	Leukocytes	SAMN21035744	4.28	6.55
	Plaque	SAMN21035752	3.54	5.78

#100	Leukocytes	SAMN21035745	3.11	5.13
	Plaque	SAMN21035753	3.70	5.93

#102	Leukocytes	SAMN21035746	3.81	6.21
	Plaque	SAMN21035754	3.76	6.10

#122	Leukocytes	SAMN21035747	3.56	5.91
	Plaque	SAMN21035755	3.62	5.94

* Sum of both reads compressed in *.bz2 archives.

Germline single-nucleotide polymorphisms (SNPs) and small indels detected in white blood cells and carotid atherosclerotic plaques of patients with atherosclerosis are stored in the Mendeley Data (doi:10.17632/hj68dfm5sm.1) in VCF file format. The data are not filtered. A brief description of this dataset is presented in Table 2. In total 1 281 674 genetic variants were called, 199 034 of them are small indels.

**Table 2:** Data on germline SNPs and small indels in patients with advanced carotid atherosclerosis.

Patient ID	Tissue type	No. of SNPs	No. of indels	Total
#46	Leukocytes	548 820	104 460	653 280
	Plaque	597 458	114 251	711 709

#57	Leukocytes	622 226	119 302	741 528
	Plaque	598 609	114 286	712 895

#60	Leukocytes	617 554	118 088	735 642
	Plaque	614 720	118 214	732 934

#67	Leukocytes	612 646	117 459	730 105
	Plaque	602 540	115 580	718 120

#95	Leukocytes	622 315	118 600	740 915
	Plaque	593 732	113 594	707 326

#100	Leukocytes	573 712	108 942	682 654
	Plaque	600 774	114 188	714 962

#102	Leukocytes	599 346	113 907	713 253
	Plaque	595 767	113 256	709 023

#122	Leukocytes	591 789	112 976	704 765
	Plaque	597 118	114 173	711 291

Somatic SNPs and small indels were deposited in the Mendeley Data (doi:10.17632/hj68dfm5sm.1) in the same VCF file format. The data are not filtered. A brief description of this dataset is presented in Table 3.

**Table 3:** Data on somatic SNPs and small indels in patients with advanced carotid atherosclerosis.

Patient ID	Filter	No. of SNPs	No. of indels	Total
#46	All	2089	1465	3554
	PASS	57	5	62

#57	All	2397	1608	4005
	PASS	47	2	49

#60	All	2452	1734	4186
	PASS	58	3	61

#67	All	2316	1657	3973
	PASS	43	6	49

#95	All	2518	1573	4091
	PASS	58	6	64

#100	All	3206	1507	4713
	PASS	61	5	66

#102	All	2447	1603	4050
	PASS	35	10	45

#122	All	2194	1483	3677
	PASS	37	7	44

## 2 Experimental Design, Materials and Methods

Matched white blood cells and a carotid atherosclerotic plaque were collected from each patient with carotid atherosclerosis (eight patients, Slavic males, aged 67 ± 3.8 years [mean ± SD]). The specimens of atherosclerotic plaques were obtained during planned carotid endarterectomy. All patients had carotid artery stenosis of more than 90%. Tissue biopsies were frozen and stored in nitrogen prior to DNA extraction.

Total-genomic-DNA samples were extracted from the white blood cells and carotid atherosclerotic plaques by means of the DNeasy Blood and Tissue Kit (Qiagen) as per manufacturer's protocol. The specimens of atherosclerotic plaques before the DNA extraction were briefly washed separately in a buffer that consisted of 0.05% of 2-mercaptoethanol, 0.5 M EDTA pH 8.0, and 1 × PBS. After that, the specimens were shredded using a disposable scalpel. This step helps proteinase K and the lysis buffer from the kit to work properly and minimizes the risk of rapid DNA degradation.

Library preparation was done using the SureSelect XT2 (SSXT2) Reagent Kit and the SureSelect Clinical Research Exome V2 Exome Enrichment Kit (Design ID S06588914), according to the manufacturer's protocol (Agilent Technologies). The quality and concentration of DNA libraries were assessed on a Qubit 3.0 instrument (Thermo) and TapeStation 2200 (Agilent Technologies). Sequencing was carried out on the Illumina HiSeq 1500 platform in 2  ×  150 bp paired-end format.

Alignments to the hg19 reference genome were conducted by the BWA-MEM algorithm [1]. The germline and somatic SNPs and indels were called by Best Practices Workflows included in the Genome Analysis Toolkit (ver. 4) [2]. Somatic SNPs and indels were identified via a comparison of exome sequences of the leukocytes and carotid atherosclerotic plaque for each patient. Statistics were analyzed by means of vcftools [3] and bcftools [4].

## Ethics Statements

The study protocol was approved by the Ethical Committee of Research Institute of Cardiology (approval number: 203). Informed consent was obtained from all patients involved in the experiments.

## CRediT authorship contribution statement

**Alexei A. Sleptcov:** Data curation, Investigation, Supervision, Writing – original draft. **Alexei A. Zarubin:** Software, Validation. **Polina M. Bogaychuk:** Software, Formal analysis. **Mikhail S. Kuznetsov:** Resources. **Boris N. Kozlov:** Data curation. **Maria S. Nazarenko:** Conceptualization, Writing – review & editing.

## Declaration of Competing Interest

The authors declare that they have no known competing financial interests or personal relationships that could have appeared to influence the work reported in this paper.
